# Association Mapping of Seed Quality Traits Under Varying Conditions of Nitrogen Application in *Brassica juncea* L. Czern & Coss

**DOI:** 10.3389/fgene.2020.00744

**Published:** 2020-09-01

**Authors:** Javed Akhatar, Mohini Prabha Singh, Anju Sharma, Harjeevan Kaur, Navneet Kaur, Sanjula Sharma, Baudh Bharti, V. K. Sardana, Surinder S. Banga

**Affiliations:** DBT Centre of Excellence on Brassicas, Department of Plant Breeding and Genetics, Punjab Agricultural University, Ludhiana, India

**Keywords:** Indian mustard, seed oil, seed protein, glucosinolates, genome-wide association study, SNP, quantitative trait loci

## Abstract

Indian mustard (*Brassica juncea*) is a major source of vegetable oil in the Indian subcontinent. The seed cake left after the oil extraction is used as livestock feed. We examined the genetic architecture of oil, protein, and glucosinolates by conducting a genome-wide association study (GWAS), using an association panel comprising 92 diverse genotypes. We conducted trait phenotyping over 2 years at two levels of nitrogen (N) application. Genotyping by sequencing was used to identify 66,835 loci, covering 18 chromosomes. Genetic diversity and phenotypic variations were high for the studied traits. Trait performances were stable when averaged over years and N levels. However, individual performances differed. General and mixed linear models were used to estimate the association between the SNP markers and the seed quality traits. Population structure, principal components (PCs) analysis, and discriminant analysis of principal components (DAPCs) were included as covariates to overcome the bias due to the population stratification. We identified 16, 23, and 27 loci associated with oil, protein, and glucosinolates, respectively. We also established LD patterns and haplotype structures for the candidate genes. The average block sizes were larger on A-genome chromosomes as compared to the B- genome chromosomes. Genetic associations differed over N levels. However, meta-analysis of GWAS datasets not only improved the power to recognize associations but also helped to identify common SNPs for oil and protein contents. Annotation of the genomic region around the identified SNPs led to the prediction of 21 orthologs of the functional candidate genes related to the biosynthesis of oil, protein, and glucosinolates. Notable among these are: *LACS5* (A09), *FAD6* (B05), *ASN1* (A06), *GTR2* (A06), *CYP81G1* (B06), and *MYB44* (B06). The identified loci will be very useful for marker-aided breeding for seed quality modifications in *B. juncea*.

## Introduction

Crop Brassicas are comprised of six economically important species belonging to the family *Brassicaceae*. These plants are cultivated as vegetables, fodder, edible oilseeds, or biofuel crops. Of these, Indian mustard (*B. juncea* L. Czern & Coss) is the most widely cultivated oilseed crop in India, with very high acreage (6.9 million hectares) and production (7.2 million metric tonnes) (USDA, 2018–2019). It is also cultivated in China, Southern Russia, and the Caspian steppes as a condiment, vegetable, and oilseed crop. *B. juncea* is an allotetraploid (AABB, 2n = 36) and it evolved through multiple hybridization events between *B. rapa* (AA, 2n = 20) and *B. nigra* (BB, 2n = 16) (Nagaharu, 1935). Breeding programs in this crop are focused on improving productivity and seed quality. Oil, protein, and glucosinolates (GSLs) determine seed quality in Indian mustard. The oil content in brassicas range from 45 to 50% and is mainly made up of unsaturated fatty acids (Smooker et al., 2011). Palmitic (C16:0), stearic (C18:0), oleic (C18:1), linoleic (C18:2), linolenic (C18:3), eicosenoic (C20:1), and erucic (C22:1) are the key fatty acids in this crop (Abbadi and Leckband, 2011). Breeding programs emphasize on providing varieties with both high (for industrial applications) and low (suitable for human consumption) erucic acid content (Beare-Rogers et al., 1971; Kaur et al., 2019). High oleic acid, low linoleic acid, or a combination of these have been developed in rapeseed-mustard crops (Appelqvist, 1971). Complex biochemical metabolic reactions with corresponding enzymes in fatty acid biosynthesis have been reviewed by Barker et al. (2007). *FAE1* plays a key role in the synthesis of erucic acid (James and Dooner, 1990) and two orthologs – *BnaA.FAE1.a* and *BnaC.FAE1.a –* are present in *B. napus*, as confirmed by associative transcriptomics (Wu et al., 2008; Harper et al., 2012; Havlickova et al., 2018). A high amount of protein is stored in the seeds of *B. juncea*, and it is reported to be negatively correlated with the oil content (Grami and Stefansson, 1977). Seed cake left after the extraction of oil is used as livestock feed. Two classes of seed storage proteins are prevalent: legumin-type globulins (11S or 12S or cruciferin) and napin-type albumins (2S or napins) (Wanasundara, 2011). The primary structural proteins of *Brassica* oilseeds are oleosins or oil body proteins. Considerable metabolic proteins, such as lipid transfer proteins (LTP), protease inhibitors (Ceciliani et al., 1994), Ca^2+^ dependent-calmodulin binding proteins (Neumann et al., 1996), and dehydrins (Svensson et al., 2000), are well-documented in *Brassica* seeds. Extensive knowledge of the protein structure, structure-function relationships, and genetic control is important for optimal utilization of *Brassica* proteins and for developing new protein-based applications.

Glucosinolates (β-thioglucoside-*N*-hydroxysulfates), a endogenous allelochemical group and a subset of secondary metabolites, are only present in the family *Brassicaceae* (Zhang and Hamauzu, 2004). Glucosinolates are anticancer, antibacterial, anti-fungal, anti-oxidative, and allelopathic compounds (Latté et al., 2011). However, these are considered anti-nutritional components in food as they can affect the thyroid function (Walker and Booth, 2001). Therefore, it is advisable to reduce GSL content in the seeds, and maintain high GSL content in other tissues to prevent herbivore damage and pathogenic microbes. Over 200 GSLs are known in *Brassica* crops. Depending on the precursor amino acid involved, glucosinolates are categorized into aliphatic, aromatic, and indole types (Fahey et al., 2001; Clarke, 2010). Glucoiberin, progoitrin, epiprogoitrin, glucoraphanin, sinigrin, glucoraphenin, gluconapin, glucobarbarin, glucobrassicanapin, glucoerucin, glucobrassicin, and gluconasturtin are the most prevalent GSLs (Wittstock and Halkier, 2002). *B. juncea* contains significant amounts of aliphatic glucosinolates (Yadav and Rana, 2018). Most of the genes responsible for biosynthetic steps are now known in *Arabidopsis thaliana* (Sonderby et al., 2010). A group of R2R3 MYB transcription factors belonging to a single gene family within *Arabidopsis* is involved in the direct transcriptional regulation of GSLs’ biosynthesis (Sonderby et al., 2010; Frerigmann and Gigolashvili, 2014). Also, MYC2, MYC3, and MYC4 regulate glucosinolate biosynthesis by directly interacting with glucosinolate-related MYB24. Limited knowledge is available for the chain-elongated homophenylalanine-derived aromatic GSLs (Bhandari et al., 2015). Genes controlling aromatic GSL biosynthesis are still uncharacterized. An extensive study and regulation of GSL natural variations in *Brassica* requires a better understanding of its genetic system. All these traits are under the control of complex regulatory mechanisms, strongly affected by the environment. So, phenotyping in replicated and multi-environment trials are important (Becker and Léon, 1988; Wang et al., 2015). Quantitative trait loci (QTL) control complex traits in crop plants and these are better understood by genome wide association studies (GWAS). Associations between phenotypes and markers can be deduced from linkage disequilibrium (LD). GWAS requires the use of numerous genome-wide markers and it provides high mapping resolution by exploiting ancestral recombination events present in a plant species (Soto-Cerda and Cloutier, 2012). It is an alluring approach for the discovery of QTLs in plant genomes associated with phenological, morphological (Honsdorf et al., 2010; Akhatar and Banga, 2015), and seed quality traits (Li et al., 2014). Many studies have been conducted to understand the genetics underlying variations for oil, protein, and glucosinolate contents in *B. napus* and *B. rapa*. However, previous studies were primarily focused on phenotypic evaluations in greenhouse or growth chambers under uniform fertilizer applications. So, there is always a theoretical possibility of missing important loci if the crop growth conditions fail to include limiting environmental variables such as Nitrogen (N), a major limiting factor for *Brassica* production (Danesh-Shahraki et al., 2008). There is a positive correlation between soil N level and seed quality (Ahmadi and Bahrani, 2009). Both protein and oil content are impacted strongly by nitrogen availability as the plants produce particular stress proteins in response to sub-optimal conditions (Bettey and Finch-Savage, 1996). For the present studies, we phenotyped 100 genotypes of *B. juncea* under real farm conditions. The crop was sown under optimal versus limited N fertilization over 2 years to generate N stress and G × N interactions. We then carried out GWAS to investigate the genetic architecture of seed quality-related traits and their stability across environments.

## Materials and Methods

### Plant Material, Field Trial, and Phenotyping

*B. juncea* diversity fixed foundation set (*Bj*DFFS), comprising 100 accessions (S_7_/S_8_ inbred lines), constituted the experimental materials for the present investigations. These accessions were evaluated in the field trials conducted during the winters of 2015–2016 (Y1) and 2016–2017 (Y2) as per the alpha lattice design with two doses of nitrogen (N) application (N0: no added N and N100: added N @100 kg/ha). The experiment was conducted in the oilseeds research area, Punjab Agricultural University, Ludhiana, India (30.9010° N, 75.8573° E). Nitrogen was added in the form of urea (46% N). For N100 treatment, urea was applied in two split doses – half at the time of sowing and the remaining half at the stem elongation stage.

Oil, protein, and glucosinolates content in intact seeds were estimated by Near Infrared Reflectance Spectroscopy (NIRS), Model 6500 spectrophotometer, Foss-NIR Systems, Inc., Silver Spring, MD, United States. Existing NIRS-based calibration models (Sen et al., 2018) were used to estimate seed quality parameters. All the trait values were adjusted for seed moisture content. We used an average of five readings per replication for each test trait.

### Statistical and Correlation Network Analysis

Statistical software SAS v9.3 was used for analyses of variance (ANOVA) to assess the significance of variance due to genotype, replication, years, N-levels, and all possible interactions between these (year × genotype, N-level × genotype, year × N-level and replication × N-level). The following model was used for this purpose.

$$P_{i\,j\,k\,l}=\;\,\mu +G_{i}+R_{j}+Y_{k}+N_{l}+{\left(Y\times G\right)}_{k\,j}+{\left(N\times G\right)}_{l\,j}$$

$$+{\left(Y\times N\right)}_{k\,l}+{\left(R\times N\right)}_{j\,l}+e_{i\,j\,k\,l}$$

where *P*_*ijkl*_ is the phenotypic value of accession, *i* noted by the *l*th N-level in year *k*, μ the overall mean, *G*_*i*_ the effect of the accession *i*, R*_*j*_* the effect of replication *j*, *Y*_*k*_ the effect of year *k*, N*_*l*_* the effect of N-level *l*, (Y × G)*_*kj*_* the interaction term between year *k* and genotype *j*, (N × G)*_*lj*_* the interaction between N-level *l* and genotype *j*, (Y × N)*_*kl*_* the interaction between year *k* and N-level *l*, (R × N)*_*jl*_* the interaction term between replication *j* and N-level *l*, and e*_*ijkl*_* the random independent and identically distributed residual term. This approach was also used for characterizing the influence of the N-levels on the trait observations: multi-year and within-year models through the use of linear mixed models. Best unbiased linear predictors (BLUPs) were assessed to obtain datasets across the years and N-levels. These estimates and predictors extracted from both year and both N-level models were then used as input data for GWAS. Pairwise Pearson’s correlation coefficients (*r*) between the traits were estimated by using the R package “Hmisc”^1^. We visualized *r* network value with the R package “q-graph.”

### SNP Genotyping

Total genomic DNA was extracted from young leaves of 100 genotypes using a standard genomic DNA extraction procedure (Doyle and Doyle, 1990), with minor modifications. DNA samples were quantified by visual comparison to λ-DNA standards on ethidium bromide-stained agarose gels. The purity and concentration of the samples were calculated using a spectrophotometer at 260 and 280 nms. High quality DNA samples were genotyped by sequencing (GBS) on the Illumina^®^ HiSeq platform, which was outsourced to Novogene (HK) Company Limited, Hong Kong. GBS data was carried out for only 92 genotypes, as DNA from the remaining eight samples failed the quality test. SNP data calling was carried out using NGSEP (Next Generation Sequencing Experience Platform) GBS pipeline^2^ (Duitama et al., 2014). *B. juncea* reference genome v.1.5^3^ was used for aligning 25× whole genome sequence of a commercial *B. juncea* genotype, PBR357, using the software Bowtie2^4^. We preferred to use a mock-up pseudomolecule reference based on the oilseed type mustard cultivar as the available *B. juncea* reference genome assembly was based on a Chinese vegetable mustard genotype, Tumida. To construct a mock-up pseudomolecule reference, total SNPs were replaced in the reference genome using a perl script, PseudoMaker, as implemented in SEG-Map (Zhao et al., 2010). All 92 inbred lines were then aligned using the pseudomolecule genome reference and, using NGSEP-GBS pipeline, SNPs were identified. SNPs with a minor allele frequency (MAF) of >0.05, minimum allele proportion of 0.7, and minimum quality score of 30 were selected for GWAS. After this filtration, a total of 66,835 SNPs were left for association studies. These are uniformly distributed over 18 chromosomes. Imputation of SNPs was performed using fcGENE v1.0.7 (Roshyara and Scholz, 2014) and BEAGLE v3.3.2 (Browning and Browning, 2007). We used R package “GAPIT” (genome association and prediction integrated tool) to transform SNP data into a numeric format (Lipka et al., 2012).

### Covariates Analysis

Different covariates, i.e., population structure, principal components (PCs) analysis, and discriminant analysis of principal components (DAPCs), are often used to overcome the bias due to the population stratification on outcomes from GWAS. These allow for adjustments for population stratification. Population structure analysis was performed by using STRUCTURE v2.3.4 (Evanno et al., 2005), with the subgroups (K) ranging from 1 to 10. The Markov Chain Monte Carlo (MCMC) repetitions were set to 10,000. An optimum number of subgroups (Q) was selected based on the log probability of the data [lnP(D)] and *ad hoc* statistic ΔK method. R package, GAPIT was used for the selection of the number of significant PCs with the largest eigenvalues based on all pairs of SNPs. Discriminant Analysis of Principal Components (DAPC) analysis was also used to reduce false positives by reducing the effects of population stratification which were implemented in the R package “adegenet.”

### Genome-Wide Association Analysis Based on SNP Genotyping

Data for protein, oil, and glucosinolates content were normalized by arcsine transformation. Genome-wide association analysis was performed on normalized values of 92 genotypes and 66,835 SNPs. Phenotypic data were pooled across 2 years for N0, N100, and NP (average values of N0 and N100) levels. Kinship matrix data was generated using the MVP-package of the software R. Q, PCs, and DAPCs analysis were used as covariates in different GWAS analysis algorithms to reduce false positives by reducing the effects of population stratification. Marker trait associations (MTA’s) were estimated by MVP (A Memory-efficient Visualization-enhanced and Parallel-accelerated Tool)^5^ with default settings to identify marker trait associations. Three methods (GLM, MLM, and FarmCPU) were used with Q, PCs, and DAPCs as covariates. Quantile-Quantile (Q-Q) plots were performed with –log_10_(*P*) of each observed SNP and the expected *P* value. These allowed us to select best fit algorithm and only corresponding SNPs were retained for further analysis. The significance of the association between SNPs and traits was assessed based on an arbitrary threshold of –log_10_(*P*) ≥ 3.

### Functional Annotation and Linkage Disequilibrium (LD)

25 kb region, upstream or downstream of peak SNPs were used for gene finding. To facilitate that, we used the software Blast2GO Pro (Götz et al., 2008) to BLAST against *A. thaliana* database. Annotation allowed us to identify candidates in the vicinity of peak SNPs. SNPs, common across N treatments, were also depicted. LD was estimated between annotated SNPs, calculating the square value of correlation coefficient (*r*^2^) between all pairs of markers by software TASSEL v5.2 (Bradbury et al., 2007) with LD type “sliding window” and LD window size “50.”

### Haplotype and Linkage Disequilibrium Analysis

Haplotypes were generated from the annotated SNPs. The haplotype and linkage disequilibrium (LD) analysis was carried out by using the software Haploview v4.2 (Barrett et al., 2005). It uses an expectation maximization (EM) algorithm to calculate measures of LD and create a graphical representation of block definitions (Gabriel et al., 2002) to partition the region into segments of strong LD.

### Meta-Analysis

The meta-analysis was conducted to test for differences in trait associations between two N-levels and 2 years. This test is based on comparing the differences between the two regression coefficients. We used the meta-analysis software METASOFT26^6^. The diversity panel was first stratified by using software PLINK v1.9 (Purcell et al., 2007). The genome-wide annotated significant SNPs *P*-values from an association test of PLINK results were used in a Binary Effects (BE) model, optimized to detect associations when some studies have an effect and others do not have any effect (Han and Eskin, 2012). ForestPMPlot, an open-source python-interfaced R package, was used to analyze the heterogeneous studies in the meta-analysis by visualizing the effect size differences between N-levels and years. The resulting plot(s) facilitated a better understanding of the heterogeneous genetic effects on the phenotypes at the different N-levels.

## Results

### Analysis of Variation for Seed Quality Traits

**FIGURE 1:**
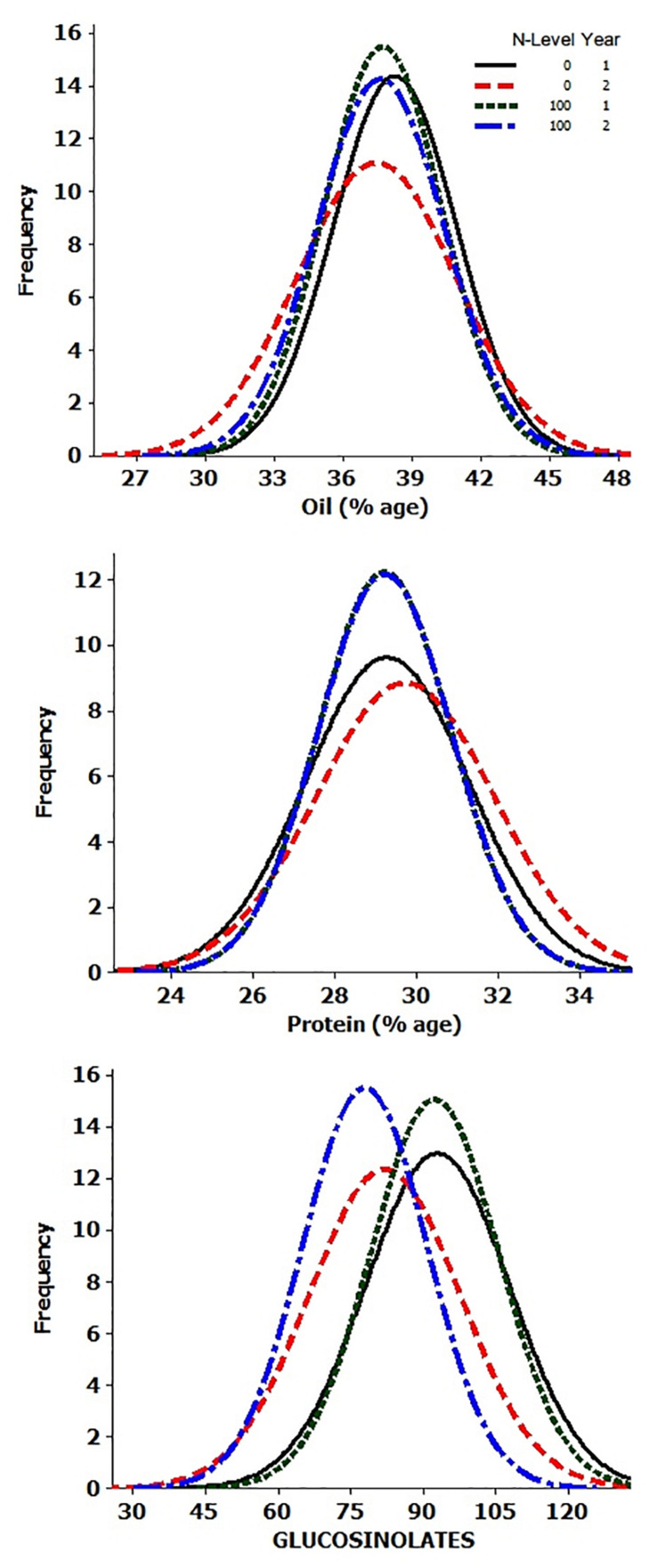
Frequency histograms of 92 genotypes for oil, protein, and glucosinotes.

Analysis of variance showed significant differences for the genotypes, years, and N-level × genotype interactions for OIL, PTN, and GSLs (Supplementary Table 1). ANOVA also revealed highly significant differences in PTN and GSLs over N levels. Variation due to Y × N interactions was significant for oil content alone. The summary data for oil, protein, and glucosinolates contents are presented in Table 1 and Figure 1. Average values for OIL remained stable over N levels and years. However, individual performances varied for both OIL and PTN contents over N and years. DJ-1-2 DT5, PLM-4, and DJ-27 DTA18 showed high OIL (≥43%) at N0Y1. In comparison, DJ-1-2 DT5 and JC-1359-23-558 accumulated more OIL during N0Y2. T-26-15C-R1631 and JC-1359-23-558 performed better for N100Y1. The same was true for JLM-96 and JC-1359-23-558 at N100Y2. Heritability (H^2^) values were generally high, but these were lower during Y1 as compared to Y2 at both N levels. PTN ranged from 23 to 35% over environments. TM117 (35%) and MCP-12-227 (34%) revealed maximum seed PTN for N0Y1, while MCP-12-227, TM117, DT 70, and PBR-357 had higher PTN (≥34%) for N0Y2. DJ-1-2 DT2 and BAUSAM-2 had high PTN (≥34%) at N100Y1. In comparison, DJ-1-2 DT2 and TM117 had maximum PTN (≥33%) at N100Y2. H^2^ for PTN ranged from 35 to 86% over years and N-levels. H^2^ was lower during Y1 as contrasted to Y2 at both N-levels. Average GSL values varied and these were lower by∼11 and 14 μmol/g during Y2 compared to Y1. The lowest GSL values were noted for the genotypes, JM-06006 (38 μmol/g) and JM-06026 (27.7 μmol/g) for N0Y2 and N100Y2 respectively. H^2^ values were lower at N100 compared to N0. Similar to OIL and PTN, H^2^ values for GSLs were lower for Y1 as compared to Y2. Oil and PTN were inversely correlated. GSL and PTN were positively associated at N0 (Supplementary Table 2 and Supplementary Figure 1).

**TABLE 1:** Descriptive statistics of the quality traits in *B. juncea* diversity panel during years 2015–2016 and 2016–2017 at two N-level.

**Trait**	**Values**	**N0**		**N100**	
		**Y1**	**Y2**	**Y1**	**Y2**
Oil (%)	Mean ± SE	38.3 ± 0.2	37.4 ± 0.4	37.7 ± 0.3	37.6 ± 0.3
	Range	(30.6–43.7)	(26.1–44.5)	(29.7–44.0)	(29.5 to 48.1)
	CV	7.0	9.3	6.8	7.4
	H^2^ (%)	44.68	91.27	47.51	63.09
PTN (%)	Mean ± SE	29.3 ± 0.2	29.7 ± 0.2	29.2 ± 0.2	29.2 ± 0.2
	Range	(25.0–34.8)	(25.2–34.4)	(25.5–33.8)	(23.0–33.1)
	CV	6.9	7.4	5.6	5.6
	H^2^ (%)	53.36	85.56	34.74	54.17
GSL (μmolg^–1^)	Mean ± SE	93.1 ± 1.5	82.1 ± 1.6	92.4 ± 1.3	78 ± 1.3
	Range	(45.5–130.1)	(38.0–125.3)	(42.8–124.5)	(27.7–107.9)
	CV	16.0	19.1	14.3	16.5
	H^2^ (%)	60.24	95.39	16.15	18.34

*N0, with no added nitrogen; N100, with added nitrogen @100 kg/ha; Y1, year 1 (2015–2016); Y2, year 2 (2016–2017); SE, standard error; CV, coefficient of variation; PTN, proteins; GSL, glucosinolates; H^2^, broad sense heritability.*

### GWAS Analysis

We used Q-Q plots to identify a best fit model (Supplementary Figure 2).

#### Oil

**FIGURE 2:**
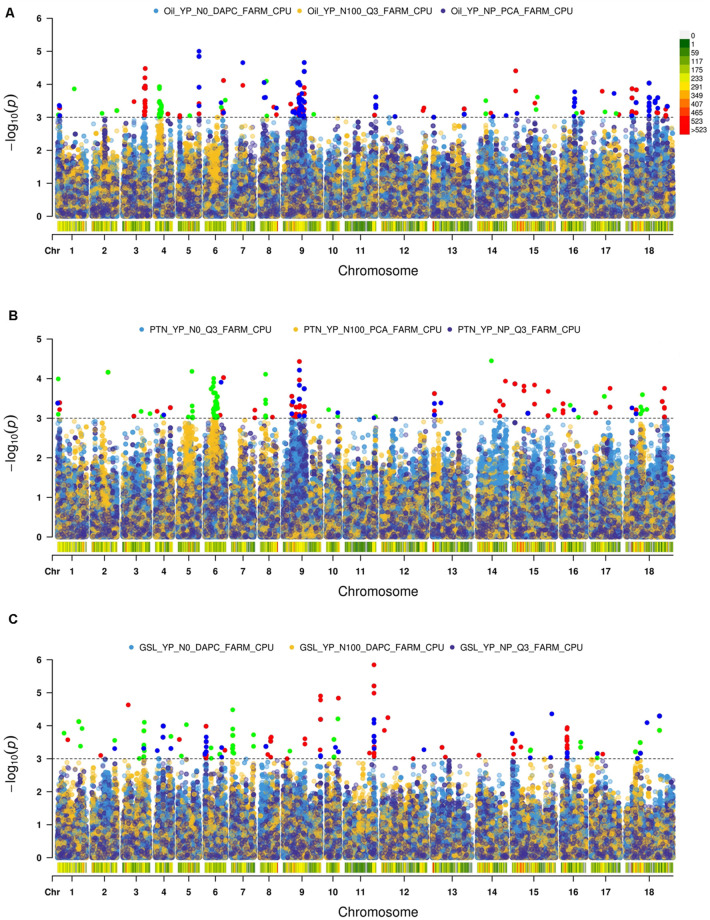
Manhattan plots for Association Analysis of **(A)** oil, **(B)** protein, and **(C)** glucosinolates.

We recognized fifteen MTA’s involving chromosomes A04, A06, A09, B05, B06, and B08 (Table 2 and Figure 2). The phenotypic variation explained by these loci ranged from 8.82 to 20.37%. Functional annotation predicted seven genes between 0.38 and 13.96 kb on either side of the peak SNPs. We envisaged *CER26* at a distance of 2.08 kb from the SNP A04_6252658. Annotation also called GDSL lipase gene at a distance of 13.96 kb from a group of three SNPs (A06_26456373, A06_26456397, and A06_26456466). This gene was also envisaged close (0.38 kb) to A09_14737522. Another gene, *LACS5* (*long-chain acyl-coA synthetase 5)*, encoding a long-chain-fatty-acid-CoA ligase, was identified near SNPs A09_24082148, A09_24082198, and A09_24083336. We annotated *FAD6* at a distance of 1.64 kb from the nearest of the associated SNPs: B05_5272794, B05_5272795, and B05_5281484. *At1g06090*, a gene encoding delta-9 desaturase-like 1 protein (SNP in the intron), and *ADS1* (2.31 kb) were called on chromosome B06 (B06_19315729). GWAS also allowed for recognition of four SNPs (B08_34645905, B08_34645977, B08_34645991, and B08_34646006) for association with *DIR1*, a gene encoding bifunctional inhibitor/lipid-transfer protein/seed storage 2S albumin superfamily protein, annotated at a distance of 3.83 kb from peak SNP.

**TABLE 2:** Summary of significant SNPs observed for quality traits of the diversity panel in GWAS and meta-analysis.

**Trait**	**Candidate gene (distance from peak SNP in kb)**	**SNP IDs**	**NCBI ID**	**Chr.**	**Position**	**GWAS**				**Meta-analysis**	
						**−log_10_(*P*)**	**PVE%**	**A.E.**	**MAF**	**Meta *P* value**	**N-level**
Oil	*CER26* (2.08)	A04_6252658	15236357	A04	6252658	3.09	14.82	1.04	0.18	0.00 × 10^–35^	N100
	GDSL esterase/lipase gene (13.96)	A06_26456373, 97, 466	15228051	A06	26456373-6466	3.17	16.29	0.47	0.17	4.33 × 10^–6^	N0, NP
	GDSL esterase/lipase gene (0.38)	A09_14737522	15228051	A09	14737522	3.19	17.1	-1.12	0.3	2.44 × 10^–6^	N0, NP
	*LACS5* (within gene, in intronic region)	A09_24082148, 2198, 3336	1032284411	A09	24082148-3336	3.39	15.88	0.45	0.3	1.44 × 10^–6^	N0, NP
	*FAD6* (1.64)	B05_5272794, 95	15235766	B05	5272794-2795	4.1	20.37	0.82	0.09	3.63 × 10^–6^	N0, NP
	*At1g06090* (within gene, in intronic region) *ADS1 (2.31)*	B06_19315729	1032298998 15221393	B06	19315729	3.03	11.92	-0.93	0.12	1.67 × 10^–7^	N0, N100, NP
	*DIR1* (3.83)	B08_34645905, 5977, 5991, 6006	18413820	B08	34645905-6006	3.34	8.82	-0.34	0.4	4.92 × 10^–5^	N0, NP
PTN	*PASTICCINO 1* (1.46)	A04_12420732, 5239	3080740	A04	12420732-5239	3.08	16.73	0	0.26	1.13 × 10^–6^	N0, N100, NP
	*AtP4H3* (within gene, in exonic region)	A06_9257535, 68	18394842	A06	9257535-7568	3.74	16.11	0	0.12	9.18 × 10^–5^	N100, NP
	*ASN1* (1.08)	A06_15534622, 4718, 4750	15232775	A06	15534622-4750	3.02	14.09	-0.23	0.12	0.00 × 10^–96^	N100, NP
	*GTR2* (6.84)	A06_17974981, 5007, 5009,5010	15241927	A06	17974981-5010	3.3	15.04	0.35	0.12	0.00 × 10^–79^	N100, NP
	*At2g41640* (9.24)	A06_27490668, 71, 93	186499036	A06	27490668-0693	4.03	23.21	0.83	0.25	8.72 × 10^–6^	N0, NP
	*PSP1* (3.71)	A09_30208138	30692244	A09	30208138	3.08	15.94	-1.05	0.11	6.28 × 10^–6^	N0, NP
	*PSP1* (1.91)	B03_3166770, 6905, 6907, 6926, 6935, 6980, 7018, 7058	42571535	B03	3166770-7058	3.3	17.84	0.28	0.23	2.74 × 10^–6^	N0, NP
GSL	*HB16* (2.29) *SK1* (3.35)	A04_11732900, 2912, 2989, 3020, 3023, 3024, 3028, 3029	189303601 189303601	A04	11732900-3029	3.99	15.48	2.37	0.19	7.99 × 10^–7^	NP^
	*AT2G35450* (1.20)	A05_5070339	79594244	A05	5070339	3.08	13.17	7.37	0.05	1.34 × 10^–5^	NP^
	*CM1* (1.70)	A06_24763290	9293913	A06	24763290	3.05	13.75	2.73	0.5	6.59 × 10^–7^	NP^
	*CM1* (1.70)	A06_24773388		A06	24773388	3.34	13.86	-3.05	0.4	2.47 × 10^–7^	NP^
	*JMT* (1.92)	B03_19283625, 26	13676829	B03	19283625-3626	3.05	15.28	0	0.08	0.00 × 10^–7^	–
	*CM1* (6.51)	B04_2285588, 589, 789	18406100	B04	2285588-5789	3.11	15.53	3.38	0.04	0.00 × 10^–73^	N0^
	*LINC4* (within gene, in exonic region)	B06_8990634, 55, 70	240256486	B06	8990634-0670	3.55	17.46	-1.51	0.49	6.61 × 10^–5^	N0^
	*CYP81G1* (12.97) *MYB44* (15.71)	B06_9279577, 727, 731	30698292 1263095	B06	9279577-9731	3.19	15.88	-4.8	0.28	1.35 × 10^–5^	N0^

*^These had highest m-values and were non-significant for meta-analysis over N-levels. PTN, proteins; GSL, glucosinolates; Chr., chromosome; PVE, phenotypic variation explained; A.E., allelic effect; MAF, minor allele frequency.*

#### Protein

We found 23 MTA’s on chromosomes A04, A06, A09, and B03 (Table 2 and Figure 2). The defined phenotypic variation ranged from 14.09 to 23.21%. Functional annotation predicted seven genes within 1.08–9.24 kb from respective peak SNPs. *PASTICCINO-1 (pas1)* was anticipated at 1.46 kb from SNPs A04_12420732 and A04_12425239. This gene encodes peptidylprolyl isomerase. We also envisage *AtP4H3* with SNPs (A06_9257535 and A06_9257568) within the exon. This gene codes for procollagen proline 4-dioxygenase. The associated SNPs were recognized in the exonic region of the gene. Three significant SNPs – A06_15534622, A06_15534718, and A06_15534750 – were present at a distance of 1.08 kb from *ASN1*, a gene encoding glutamine dependent-asparagine synthetase. *GTR2* was envisioned at a distance of 6.84 kb from the closest of the associated SNPs: A06_17974981, A06_17975007, A06_17975009, and A06_17975010. *At2g41640*, encoding glycosyltransferase family 61 protein, was predicted at a distance of 9.24 kb from SNPs A06_27490668, A06_27490671, and A06_27490693. We also detected *PSP1* (*PHOSPHOSERINE PHOSPHATASE 1)* on the chromosome A09 at a distance of 3.71 kb from SNP (A09_30208138). This gene was also envisioned next to the peak SNP on the chromosome B03.

#### Glucosinolates

We identified 22 MTA’s involving chromosomes A04, A05, A06, B03, B04, and B06 (Table 2 and Figure 2). The phenotypic variation explained varied from 13.17 to 17.46%. Functional annotation predicted 10 genes in the vicinity (1.20–15.71 kb) of the significant SNPs. Of these, eight SNPs (A04_11732900, 2912, 2989, 3020, 3023, 3024, 3028, and 3029), were located close to the predicted genes *HB16* and *SK1* at the respective distances of 2.29 and 3.35 kb from the peak SNPs. Both these encode shikimate kinase, which catalyzes a step of shikimate pathway for the synthesis of aromatic amino acids (tryptophan, tyrosine, and phenylalanine). We also established a gene *AT2G35450* on chromosome A05 at a distance of 1.2 kb from SNP A05_5070339. Chorismate mutase1 (*CM1*) was predicted close to two SNPs on chromosome A06 at a distance of 1.7 kb and three SNPs for chromosome B04 at a distance of 6.51 kb from the respective peak SNPs. Two significant SNPs, B03_19283625 and B03_19283626, were associated with JMT, encoding jasmonate O-methyltransferase. Three SNPs (B06_8990634, 55, and 70) were detected within the exon of the predicted gene, *LINC4*. This gene encodes branched chain-amino acid-transaminase. *CYP81G1 and MYB44* were envisioned at distances of 12.97 and 15.71 kb from the respective significant SNPs present on chromosome B06.

### LD Plot of Annotated SNPs

**FIGURE 3:**
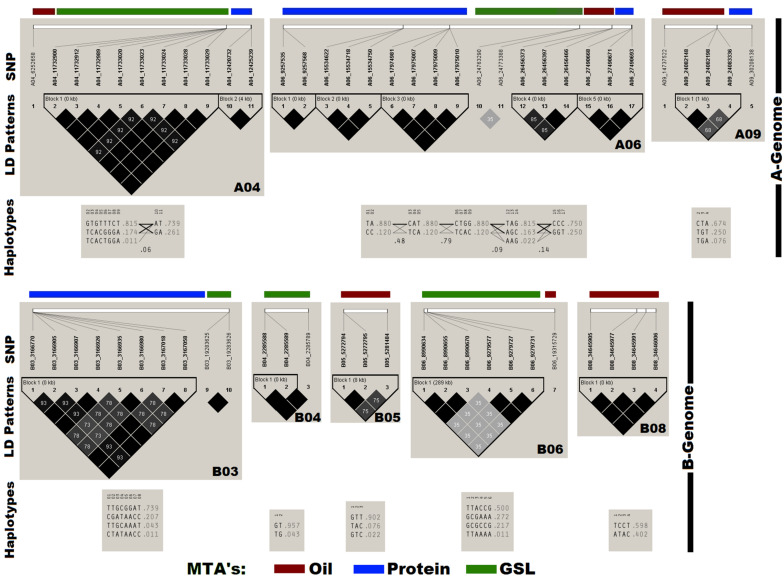
Chromosome-wise linkage disequilibrium (LD) plot generated using Haploview. The plot also depicts the haplotypes block containing the annotated SNPs for OIL, PTN, and GSL. Dark gray colors in LD plots indicate strong LD between markers as estimated by *r*^2^ values.

Haplotype analysis and pair-wise LD estimation was performed in the panel using 60 SNPs annotated for OIL, PTN, and GSL (Figure 3). Thirteen out of sixteen SNPs associated with OIL generated four haplotype blocks on chromosomes A06, A09, B05, and B08. We found six haplotype blocks on chromosomes A04, A06, and B03 for PTN with strong *r*^2^ values. The remaining 16 SNPs produced three haplotype blocks on the chromosomes A04, B04, and B06 for GSLs.

### Meta-Analysis

**FIGURE 4:**
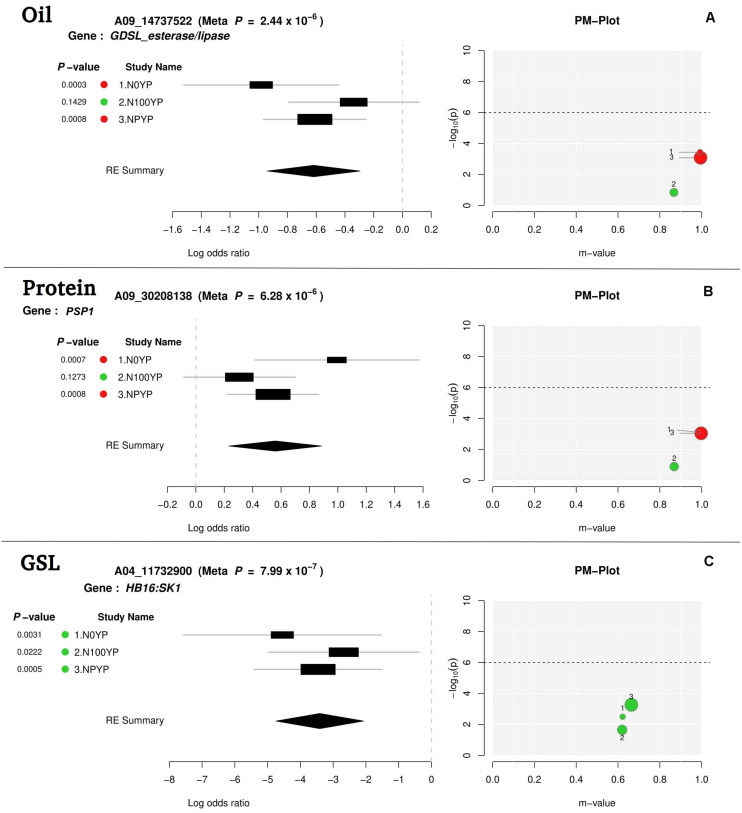
Meta-analysis output file for oil **(A)**, protein **(B)**, and glucosinolates content **(C)** (one gene for each trait).

For meta-analysis, we looked into 21 candidate genes that were significantly associated with OIL, PTN, and GSL. Meta-analysis allowed reconfirmation of most GWAS-SNPs for OIL and PTN, but with higher mapping resolution and an improved number of significant variants (over N levels) (Table 2 and Figure 4). *At1g06090* and *ADS1* were predicted repeatedly at both N levels (meta *p* = 1.67 × 10^–7^) with B06_19315729. *DIR1*, predicted on chromosome B08, was significant at both N0 and NP in meta-analysis while this was significant at NP in GWAS. For PTN (Table 2), *PASTICCINO1* was called twice on chromosome A04 at meta *p* value of 1.13 × 10^–6^. *AtP4H3*, *ASN1*, and *GTR2* (all on A06) were significant at N0 and NP following meta-analysis. These were significant at N100 alone for GWAS. SNP linked to *At2g41640*, a gene encoding glycosyltransferase family 61 protein, was significant at N0 and NP in meta-analysis (meta *p* = 8.72 × 10^–6^). It was significant only at N0 level following GWAS. *PSP1*, with A09_30208138 as associated SNP, was significant at N0 and NP during meta-analysis (meta *p* = 6.28 × 10^–6^). For glucosinolates, *HB16* and *SK1* were present close to eight SNPs associated with chromosome A04. These were significant at NP during GWAS. We recorded the highest but non-significant m values (meta-analysis) for NP (Table 2). A similar trend was observed for *CM1* envisaged on chromosome A06 twice. We also recognized *CM1* on chromosome B04 with an m-value of 10^–73^. It was significant at N0 for GWAS but non-significant following meta-analysis (highest m values at N0). Almost similar trends were recorded for *LINC4*, *CYP81G1*, and *MYB44* on the chromosome B06.

*In silico* analysis of the genome sequences of *B. napus* and *B. juncea* allowed identification of multiple copies of the genes that predicted seed quality traits on the A genome common to both *B. juncea* and *B. napus* (Table 3). A fair degree of parallelism existed, but these were differences in their location on specific A genome chromosomes.

**TABLE 3:** Details of identified gene copies in A genome.

**Trait**	**AT ID**	**Gene/protein**	**Chromosome(s)**	
			***B. juncea***	***B. napus***
Oil	*AT1G06080*	*ADS1*	A09, A10	A6, A10
	*AT1G06090*	Delta-9 desaturase-like 1 protein	A08, A09, A10	A6, A8, A10
	*AT2G03980*	*GDSL* esterase/lipase	A02, A06, A09	A2, A2, A9
	*AT4G11030*	*LACS5*	A02, A09	A6, A8
	*AT4G12510*	*DIR1*	A03	A3
	*AT4G13840*	*CER26*	A04	A4
	*AT4G30950*	*FAD6*	A01, A08	A1, A8
	*AT1G31070*	*GlcNAc1pUT1*	A05, A08, A09	A8, A9
	*AT2G03360*	Glycosyltransferase family 61 protein	A06, A09	–
	*AT3G47340*	GLUTAMINE-DEPENDENT ASPARAGINE SYNTHETASE	A02, A06	A6
	*AT3G54010*	*PASTICCINO-1*	A04, A07	A4
	*AT5G62680*	*GTR2*	A02, A03, A06, A09	A1, A2, A6, A9
GSL	*AT1G19640*	*S*-adenosyl-L-methionine-dependent methyltransferases superfamily protein	A06, A07, A08	A6, A7
	*AT2G21940*	*SK1* (AT2G21940)	A04	A6
	*AT2G35450*	AT2G35450	A05	–
	*AT3G29200*	*CM1*	A06, A09	A6, A9, A9
	*AT4G40060*	*HB16*	A01, A08	A1, A8
	*AT5G65770*	*LINC4*	A06, A09	A2, A2
	*AT5G67310*	*CYP81G1*	A07	A7, A7

## Discussion

The development of high-yielding strains with increased oil and protein contents coupled with low meal glucosinolates in seeds is a major crop improvement goal in *B. juncea*. Simultaneous improvement for these interrelated traits is, however, challenging due to complex genetics and large environmental influences (Banga et al., 2015). Many investigations have been undertaken to understand the genetics of seed oil content in *B. juncea* (Mahmood et al., 2006; Ramchiary et al., 2007; Yadava et al., 2012; Rout et al., 2018), *B. napus* (Qiu et al., 2006; Cao et al., 2010; Zhao et al., 2012; Wang et al., 2013; Jiang et al., 2014; Körber et al., 2016; Fu et al., 2017), and *B. carinata* (Zhang et al., 2017). GWAS was also combined with transcriptome analyses to predict seven functional candidate genes for the seed oil content in *B. napus* (Xiao et al., 2019). However, there are only a few studies involving association mapping for seed quality traits in *B. juncea*. We investigated the genetics of seed quality traits through GWAS based on an association panel. Trait phenotyping was carried out at two doses of N application. ANOVA revealed highly significant differences in PTN and GSLs over N levels, but OIL was relatively stable. Relatively stable expression of oil content is largely in confirmation with an earlier report in mustard (Chauhan et al., 2012). GWAS and meta-GWAS allowed us to examine environment (N) specific SNPs and candidate genes related to OIL, PTN, and GSL. We identified several genes assigned to oil content and fatty acid synthesis. These were placed on both A-(A04, A06, A09) and B-(B05, B06, B08) genome chromosomes. Notable among these were *CER26*, GDSL lipase gene, and *LACS5. CER26* has a role in fatty acid elongation (Pascal et al., 2013), while GDSL esterase/lipase (GLIP) gene(s) encode hydrolytic enzymes involved in development and morphogenesis as reported in rice, *Arabidopsis*, and maize (Akoh et al., 2004). GDSL lipase gene was first isolated from *B. napus* (Ling et al., 2006) and QTLs for esterase and lipases family protein have been reported in *B. napus* (Gu et al., 2017). *LACS5* is a floral tissue-specific gene that contributes to oil biosynthesis (Shockey et al., 2002). *FAD6* (called on chromosome B05) encodes delta-12 desaturase that aids in fatty acid desaturation (Mikkilineni and Rocheford, 2003). We envisaged delta9 desaturase (Δ9 DS) and *ADS1* on chromosome B06 at all N levels. Δ9 DS, an intrinsic membrane protein, regulates the catalytic desaturation of saturated fatty acids at the C9 and C10 positions to form unsaturated fatty acids (Stukey et al., 1990). *ADS1* has been used widely for genetically modifying saturated fatty acids in oilseed crops (Yao et al., 2003). Plant lipid-transfer proteins (LTPs) are able to reversibly bind and transport lipids *in vitro* (Kader et al., 1984). We predict gene encoding LTPs on chromosome B08 in contrast to their reported presence on chromosomes A04, A05, and A06 in *B. napus* (Wang et al., 2018). A fair degree of parallelism existed for the genes associated with oil and fatty acid identified for A-genomes of *B. juncea* and *B. napus*. Candidate genes predicted in our studies were predominantly located on chromosomes A04, A06, and A09. Chromosome A09 was also important for oil content in *B. napus* (Xu et al., 2017; Xiao et al., 2019).

Seed meal of *B. juncea* is protein-rich (Grami and Stefansson, 1977; Das et al., 2009) and it is used as livestock feed depending on its nutritional value (Wanasundara, 2011). We anticipated many genes associated with plant development, transport, and protein synthesis on chromosomes A04, A06, A09, and B03. Our *in silico* analysis also predicted corresponding orthologs for A03, A04, A06, and A09 for A genome of *B. napus*. *PASTICCINO1*, important for coordinating cell division and differentiation during plant development (Harrar et al., 2003), was envisioned on chromosome A04 at all N levels. Glutamine-dependent asparagine synthetase (*ASN1*) was envisaged on chromosome A06. It is required in nitrogen storage and transport (Canales et al., 2012). Transgenic *Arabidopsis* lines over-expressing *ASN1* produced high seed-soluble protein (Lam et al., 2003). We also identified that *GTR2* is a glucosinolate-specific transporter. It regulates the loading of glucosinolates from the apoplast into the phloem in *Arabidopsis* (Nour-Eldin et al., 2012). Two copies of a functional nucleotidyltransferase gene, *PSP1*, were annotated on chromosomes A09 and B03. *PSP1* encodes UDP-N-acetylglucosamine diphosphorylase 1, a fundamental precursor for glycoprotein and glycolipid synthesis (Yang et al., 2010).

Glucosinolates are the most extensively studied defense-related secondary metabolites, produced exclusively in the family *Brassicaceae* (Halkier and Gershenzon, 2006; Yatusevich et al., 2010). Very large numbers of genes encoding various steps of glucosinolates biosynthesis have been predicted in *B. rapa* (Wang et al., 2011) and *B. napus* (Chalhoub et al., 2014). Expression of these genes is subject to influence by temperature, nitrogen, and sulfur (Barthet and Daun, 2011). We recorded eight genes on chromosomes A04, A05, A06, B03, B04, and B06. These include *HB16* and *SK1*, which encodes shikimate kinase (SK). It is an enzyme of shikimate pathway that directs carbon from the central metabolism pool to a broad range of secondary metabolites (Fucile et al., 2008; Tzin and Galili, 2010; Tohge et al., 2013; Averesch and Krömer, 2018). *CM1*, predicted on chromosomes A06 and B04, is associated with the biosynthesis of phenylalanine and tyrosine. It also participates in tryptophan biosynthesis (Westfall et al., 2014; Qian et al., 2019). *JMT* was annotated on chromosome B03. It is critical for jasmonate-dependent induction of indole glucosinolates in *Arabidopsis* (Brader et al., 2001; Ku et al., 2016). *LINC4* was predicted on B06. It encodes branched-chain-amino-acid transaminase. This enzyme catalyzes the conversion of branched-chain amino acids and α-ketoglutarate into branched chain α-keto acids and glutamate (De Kraker et al., 2007). We identified *CYP81G1* on chromosome B06. It is involved in the metabolic processes of indole glucosinolates (Halkier, 1999). *MYB44* (*AT4G37260*) was envisioned on chromosome B06. *MYB44* regulates the expression of most GSL biosynthesis genes in partnership with *EIN2* (Lü et al., 2013). *In silico* analysis of *B. juncea* genome assembly revealed four orthologous copies (A02, A07, A09, and B06) with a coding sequence comparable to *MYB44*. Three major QTLs are known for GSL content in *B. napus* (Howell et al., 2003; Li et al., 2014; Lu et al., 2014; Qu et al., 2015). These colocalized with three orthologs of the Arabidopsis *MYB28* on chromosomes A09, C02, and C09 (Chalhoub et al., 2014; Lu et al., 2014; Wang et al., 2018).

To summarize, we identified 21 orthologs of the functional candidate genes related to the biosynthesis of OIL, PTN, and GSL. As was expected for a polyploid crop, *in silico* analysis of the reference genome sequence revealed multiple copies of predicted genes on different A- and B- genome chromosomes. We were also able to establish LD patterns and haplotype structures for the candidate genes. The average block sizes were larger on A-genome chromosomes as compared to the B- genome chromosomes. Genetic associations differed over N levels and meta-analysis of GWAS datasets not only improved the power to detect associations but also helped to identify common SNPs. N0 proved better to unravel subtle variations for OIL. In contrast, evaluation at N100 appeared suitable for investigating PTN.

## Data Availability Statement

The datasets generated for this study can be found in the NCBI under bioproject PRJNA639209.

## Author Contributions

SB developed genetic resources and designed and supervised the research, edited the manuscript. VS helped in the conduct of field trials. SS performed biochemical analysis. JA conducted statistical and bioinformatics analysis. BB helped with statistical analysis. MS and AS annotated the results. MS, AS, HK, JA, and NK interpreted the results and wrote the manuscript. All authors have read and approved the version of manuscript.

## Conflict of Interest

The authors declare that the research was conducted in the absence of any commercial or financial relationships that could be construed as a potential conflict of interest.

## Footnotes

1 http://biostat.mc.vanderbilt.edu/wiki/Main/Hmisc

2 https://github.com/NGSEP/NGSEPcore

3 http://brassicadb.org/brad

4 http://bowtie-bio.sourceforge.net/bowtie2

5 https://github.com/XiaoleiLiuBio/MVP

6 http://genetics.cs.ucla.edu/meta/

## Supplementary Material

TABLE S1 Analysis of Variance (ANOVA).

TABLE S2 Correlation (*r*) of the quality traits across years (Y1 & Y2) at two N-levels: N0 and N100.

FIGURE S1 A correlation network plot based on Pearson’s correlations (*r*) for seed quality traits across years and N-levels. Black connecting lines denote positive and red connecting lines denote negative correlations (G = GSL = Glucosinolates; P = Protein; O = Oil; N0Y1 = N-level (N0) at Year1; N0Y2 N-level (N0) at Year2; N100Y1 N-level (N100) at Year1 and N100Y2 = N-level (N100) at Year2.

FIGURE S2 Q-Q plots for selection of the best fitted GWAS algorithm.
