# Supplementary material for: Association Mapping of Seed Quality Traits Under Varying Conditions of Nitrogen Application in Brassica juncea L. Czern & Coss
Source: Front Genet. 2020 Sep 1;11:744. doi: 10.3389/fgene.2020.00744 (PMC7490339; doi:10.3389/fgene.2020.00744)
Supplement: TABLE S1 — Analysis of Variance (ANOVA). [file Data_Sheet_1.docx]

Supplementary Table 1. Analysis of Variance (ANOVA).

| **Source** | **d. f.** | **Mean Square (MS)** | | |
| --- | --- | --- | --- | --- |
|  |  | **Oil** | **Protein** | **Glucosinolates** |
| Genotype | 91 | 31.51** | 14.31** | 730.65** |
| Rep | 1 | 4.90 | 0.97 | 1,087.69* |
| Year | 1 | 40.43** | 9.41* | 29,564.06** |
| N-Level | 1 | 0.15 | 18.84** | 1,208.55** |
| Year × Genotype | 91 | 2.81 | 1.08 | 82.12 |
| N-Level × Genotype | 91 | 30.39** | 12.41** | 755.21** |
| Year × N-Level | 1 | 25.75* | 7.46 | 556.99 |
| Rep × N-Level | 1 | 81.99** | 41.72** | 284.69 |
| Error | 457 | 4.59 | 2.15 | 179.38 |

* and ** are significant levels at 5% and 1%, respectively.

Supplementary Table 2. Correlation of the quality traits at two N-levels – N0 and N100.

| **At N0** |  | **Oil** | **Protein** | **At N100** | **Oil** | **Protein** |
| --- | --- | --- | --- | --- | --- | --- |
|  | **Protein** | -0.738** |  |  | -0.750** |  |
|  | **GSL** | -0.121 | 0.212* |  | -0.01 | 0.102 |

* and ** are significant levels at 5% and 1%, respectively.
