# Supplementary figures and images for: Association Mapping of Seed Quality Traits Under Varying Conditions of Nitrogen Application in Brassica juncea L. Czern & Coss
Source: Front Genet. 2020 Sep 1;11:744. doi: 10.3389/fgene.2020.00744 (PMC7490339; doi:10.3389/fgene.2020.00744)

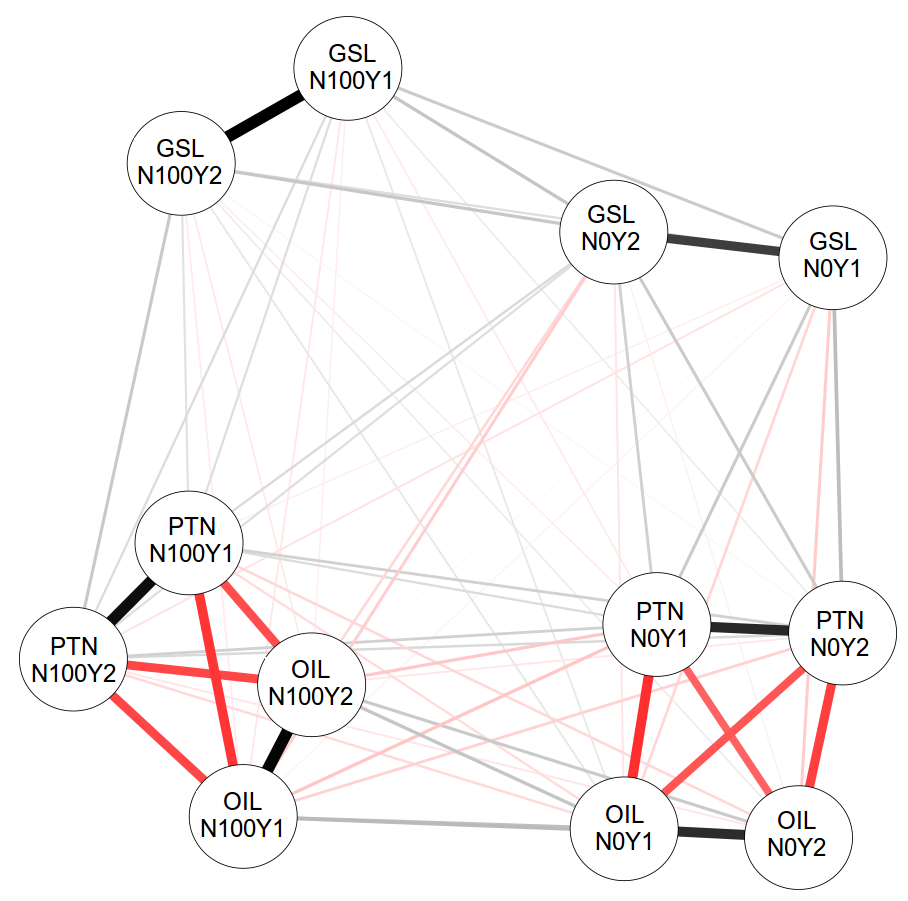

Supplement: FIGURE S1 — A correlation network plot based on Pearson’s correlations (r) for seed quality traits across years and N-levels. Black connecting lines denote positive and red connecting lines denote negative correlations (G = GSL = Glucosinolates; P = Protein; O = Oil; N0Y1 = N-level (N0) at Year1; N0Y2 N-level (N0) at Year2; N100Y1 N-level (N100) at Year1 and N100Y2 = N-level (N100) at Year2. [file Image_1.TIFF]

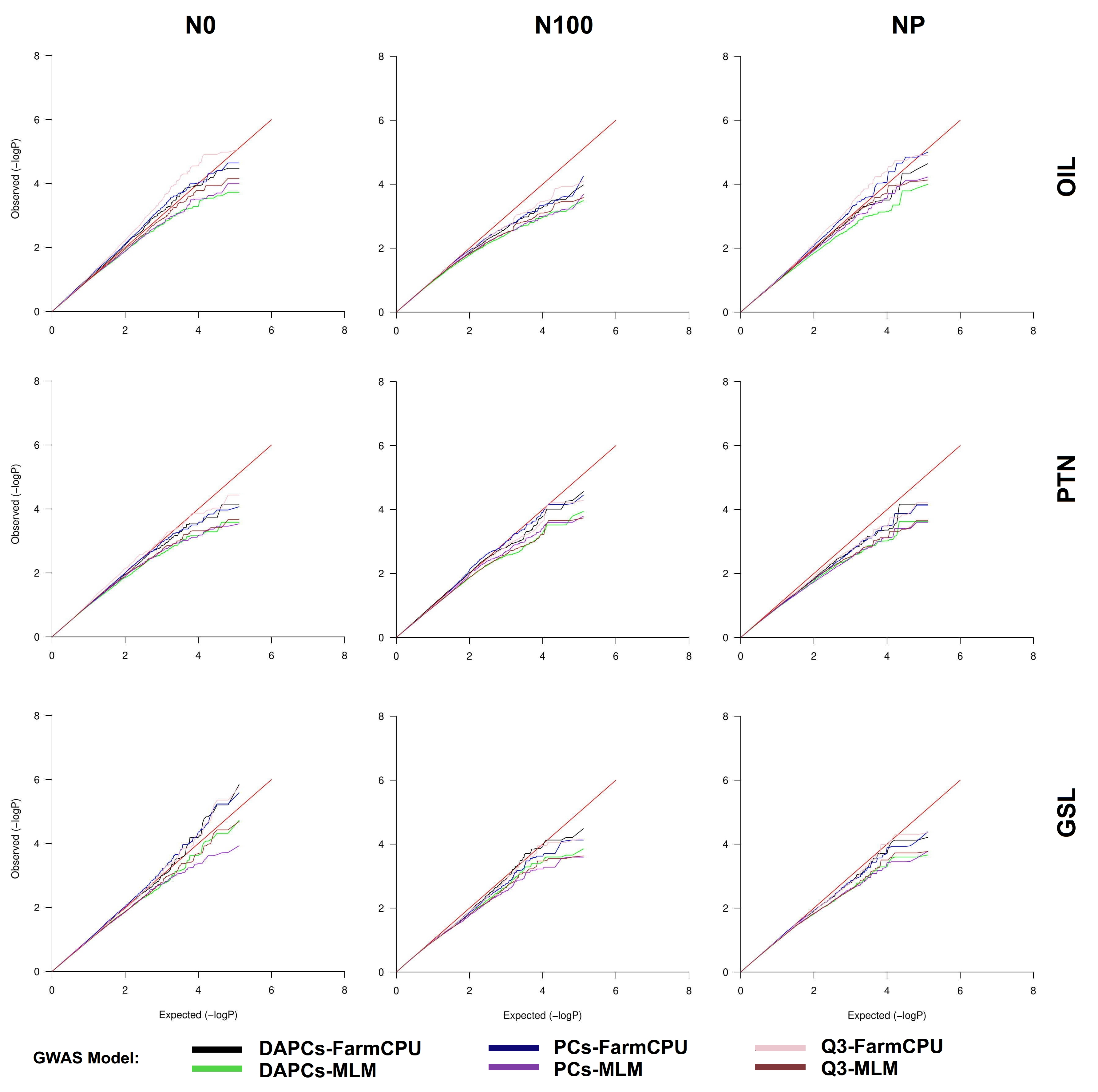

Supplement: FIGURE S2 — Q-Q plots for selection of the best fitted GWAS algorithm. [file Image_2.TIF]
